# Effects of 5-Minute Light-Intensity Physical Activity Breaks (Sit-Cycling and Stand-Twisting) on Cognitive Function, Sleepiness, and Back Pain in Physically Inactive University Students: Protocol for a Mixed-Design Laboratory Study

**DOI:** 10.2196/82510

**Published:** 2026-03-17

**Authors:** Jiameng Ma, Buan Kiong Terence Chua, You Hui Jacqueline Chin, Wei Peng Teo, Hyunshik Kim, Seow Ting Low, Dan Li, Yong Hwa Michael Chia

**Affiliations:** 1 Physical Education and Sports Science National Institute of Education Nanyang Technological University Singapore Singapore; 2 Faculty of Sports Science Sendai University Shibata-machi, Miyagi-ken Japan; 3 Science of Learning in Education Centre National Institute of Education Nanyang Technological University Singapore Singapore

**Keywords:** physically inactive, sit-cycling, stand-twisting, cognitive function, daytime sleepiness, back pain

## Abstract

**Background:**

University students may remain sedentary for large parts of the day, spending >9 hours per day sitting. Prolonged sitting is associated with increased risk for adverse health outcomes in adulthood. Light-intensity physical activity (LIPA) can be integrated into university students’ daily routines to counteract the negative effects of prolonged sitting on cognitive function and musculoskeletal health.

**Objective:**

This protocol describes a trial conducted in Singapore to evaluate the acute (immediate) and short-term delayed effects of two 5-minute LIPA modalities (sit-cycling [S-C] and stand-twisting [S-T]) on neurocognitive and somatic outcomes (prefrontal cortical oxygenation, cognitive performance, subjective sleepiness, and back pain) in physically inactive university students compared to a modality-matched passive control condition.

**Methods:**

The study comprised a main study (N=22) and a methodological substudy (n=6, 27.3%). In the substudy, students’ oxygen uptake was measured during 5-minute bouts of S-C and S-T to verify that both modalities meet the operational definition of LIPA. In the main study, physically inactive university students were allocated in a 1:1 ratio to either S-C or S-T (between-participant factor). Each participant completed two laboratory visits within their assigned modality: an experimental LIPA visit and a corresponding modality-matched passive control visit of equal duration (passive sitting for S-C and S-T), enabling within-person intervention-control comparisons. The primary endpoint is the change in bilateral dorsolateral prefrontal cortex (DLPFC) oxygenated hemoglobin (O2Hb) from baseline to immediately after the intervention during cognitive task performance (intervention vs control), measured with functional near-infrared spectroscopy (fNIRS). Secondary outcomes include O2Hb persistence at 20 minutes postintervention, cognitive performance (n-back test and Stroop Color and Word Test [SCWT]), subjective sleepiness, and back pain.

**Results:**

The study was funded by the Sendai University International Collaborative Research Grant in April 2023, and the trial was registered with ClinicalTrials.gov on November 21, 2024. Participant recruitment and data collection were completed in December 2024. The substudy results showed that both 5-minute S-C and S-T bouts fell within the LIPA range of <3 metabolic equivalents (METs) and could be implemented as brief, standardized LIPA breaks in the main trial. For the main trial, data cleaning and preliminary analyses are ongoing, and the outcomes will be reported in a subsequent outcomes paper (anticipated in late 2026).

**Conclusions:**

This protocol provides a prespecified framework to test whether brief 5-minute LIPA bouts may be a practical strategy to interrupt prolonged sitting and acutely improve prefrontal oxygenation and related neurocognitive and somatic outcomes in physically inactive university students.

**Trial Registration:**

ClinicalTrials.gov NCT06703424; https://clinicaltrials.gov/study/NCT06703424

**International Registered Report Identifier (IRRID):**

PRR1-10.2196/82510

## Introduction

### Background

It is common for university students to remain sedentary for large portions of the day, spending over 9 hours per day sitting [1]. Prolonged sitting is associated with increased risk for adverse health outcomes in adulthood, including cardiovascular disease [2], various forms of cancer, and type 2 diabetes [3]. In this context, understanding sedentary behavior among university students in Singapore is particularly important, as lifestyle patterns in a highly urbanized environment may differ from those in other regions.

Population-based studies in Singapore indicate that 47.7% of adults spend at least 7 hours per day in sedentary activities. Notably, the highest prevalence (59.8%) is observed among individuals aged 18-34 years [4], a demographic that closely aligns with the typical age range of university students. This pattern raises concerns about the potential health implications of prolonged sitting in this age group.

High levels of sedentary behavior appear to persist despite a reported increase in leisure-time sports and exercise participation among Singaporean citizens and residents, as documented in the National Sports Participation Survey [5]. This apparent paradox suggests that even when individuals engage in exercise, they may still spend substantial portions of the day sedentary, potentially undermining the health benefits of physical activity.

Meta-analytic evidence further indicates that the health risks linked to prolonged sitting are largely independent of an individual’s overall physical activity level, including among those who meet established physical activity guidelines [2,3]. Therefore, sedentary behavior should be addressed as a distinct risk factor for later ill health, rather than being considered solely in relation to insufficient physical activity.

The Movement Ecology Framework (MEF) conceptualizes human movement as a natural and essential component of health and well-being, emphasizing that even low-intensity, nonexercise movements (eg, fidgeting, stretching, or trunk-twisting) can contribute to metabolic and brain health [6]. Anchored in the MEF, this study will focus on light-intensity physical activity (LIPA) as a practical approach that can be integrated into university students’ daily routines to counteract the negative effects of prolonged sitting on cognitive function and musculoskeletal health, potentially improving sleep-related outcomes and reducing back pain [7].

Interventional studies suggest that incorporating short physical activity breaks can effectively reduce excessive sitting among university students [8]. Such interventions may mitigate risks associated with prolonged sedentary behavior and contribute to improvements in physical health [9,10], mental health [11,12], and work productivity [13,14]. These findings underscore the importance of developing strategies that encourage regular movement throughout the day.

The association between sedentary behavior and cognitive function is increasingly recognized. Neuroimaging studies have shown that prolonged sitting correlates with reduced structural thickness in brain areas, such as the medial temporal lobe, which may impair cognitive abilities [15]. Systematic reviews also suggest that sedentary behavior is associated with lower cognitive function [16,17], although some studies report weaker associations [18]. Experimental evidence indicates that short physical activity breaks may offset some negative effects of prolonged sitting by enhancing cerebral blood flow [19], particularly to the frontal lobe, which supports complex cognitive processes [20-25]. In addition, sedentary behavior may adversely affect mood and physical health, contributing to fatigue, back pain, and hypertension [26-28].

Intervention studies in older adults suggest that interrupting sitting with brief walks can increase brain-derived neurotrophic factor and improve cognitive function [29]. However, similar strategies have not consistently demonstrated significant benefits in university students, potentially due to differences in exercise intensity requirements or the relatively higher baseline physical activity levels common in this population [30-32]. Although sit-cycling (S-C) has been used as a method to interrupt prolonged sitting [33], stand-twisting (S-T) has, to the best of our knowledge, not been used as a modality for breaking up prolonged sitting.

### Research Intention

S-C and S-T are cost-effective and space-efficient LIPA modalities that can be implemented in typical university settings, making them practical strategies for increasing gross motor movement and interrupting prolonged sitting. The intervention duration in this study was set at 5 minutes to balance physiological plausibility with real-world feasibility. Brief LIPA breaks of this duration are implementable in university classrooms, libraries, and study spaces with minimal disruption to academic routines, reflecting realistic conditions under which sedentary-behavior interruptions could be adopted. Mechanistically, a short bout of light movement may elicit rapid changes in autonomic activation and cerebral perfusion/oxygenation, which may be accompanied by short-term changes in executive performance, while minimizing fatigue or musculoskeletal discomfort that could influence subjective symptom ratings.

### Study Purpose

A two-arm study using sequential (quasi-random) allocation was designed to examine the acute (immediate) and short-term delayed effects of a single 5-minute bout of either S-C or S-T on the prespecified outcomes, relative to a modality-matched 5-minute passive control (passive sitting for S-C and S-T). This is a mixed within-between laboratory study in which participants were assigned to one modality only (S-C or S-T; between-participant factor) and completed two visits within that modality—an intervention visit and a modality-matched passive control visit—constituting a two-period within-participant crossover for the condition with repeated assessments over time; intervention-control effects will be evaluated within person, whereas modality effects will be compared between participants (ie, no crossover between modalities). The study consisted of two parts: an oxygen uptake measurement phase (*substudy*) and an intervention phase (*main study*). In essence, the substudy focused on the measurement of oxygen uptake during a single 5-minute bout of S-C or S-T, while the main study examined the transient and delayed effects of a single 5-minute bout of S-C or S-T on oxygenated hemoglobin (O_2_Hb) levels in the bilateral dorsolateral prefrontal cortex (DLPFC), cognitive function, blood pressure (BP), and self-reported daytime sleepiness and back pain compared to 5 minutes of passive sitting. These passive comparators were designed to match exposure time and posture but were not intended as sham interventions.

### Standard Protocol Items: Recommendations for Interventional Trials

The study adhered to the SPIRIT (Standard Protocol Items: Recommendations for Interventional Trials) guidelines [34]. This protocol describes the design and development of the intervention study using 5-minute bouts of S-C and S-T and involves an investigation into the oxygen uptake during 5 minutes each of S-C and S-T compared to the passive rest states of sitting. Findings from this study will add knowledge to the literature about the effectiveness of novel LIPA, such as S-C and S-T, in reducing sedentary behavior and its impact on performance tasks and somatic symptoms. Future research can adapt the described protocol for application in different desk-bound or movement-restricted occupational settings in larger-scale studies (eg, for deskbound administrative staff or classroom-based schoolteachers) to interrupt prolonged occupational sitting.

### Study Objectives and Hypotheses

#### Substudy: Measurement of Oxygen Uptake in 5 Minutes of S-C and S-T and Intensity Validation

The objective of the substudy is to quantify oxygen uptake during a single 5-minute bout of S-C or S-T performed at a self-selected pace and to determine whether both modalities meet the operational definition of LIPA (<3 metabolic equivalents [METs]). We hypothesized that S-C against a negligible or minimal applied resistance and S-T at a self-determined cadence (about 2 seconds per full twist to the left and to the right) are LIPA-compliant and incur an energy output of <3 METs. Physical activities involving <3 METs are typically classified as LIPA. Data from an earlier study using the same S-C equipment showed that the intensity of S-C at a self-selected pace in older working adults is of light intensity [33]. Moreover, the use of waist-twisters at public outdoor parks are considered LIPA [35].

#### Main Study: Examining the Effect of Light-Intensity S-C and S-T

Prefrontal hemodynamic responses will be evaluated using O_2_Hb, with the primary endpoint defined as the within-person intervention-control difference in the change in bilateral DLPFC O_2_Hb levels (region-of-interest average) from baseline (t_0_) to immediately after the intervention (posttest 1, t_1_) during cognitive task performance. Key secondary outcomes include persistence of the O_2_Hb response 20 minutes postintervention (posttest 2, t_2_) and cognitive performance (n-back test and Stroop Color and Word Test [SCWT]; accuracy/response time, as applicable). Deoxygenated hemoglobin (HHb) will be analyzed as a supportive physiological indicator, and all remaining physiological and symptom-based measures have been prespecified as secondary/exploratory outcomes.

The objectives of the main study are as follows:

- To compare changes in prefrontal cortical hemodynamics (O2 Hb and HHb) between each intervention and its corresponding control across baseline (t0), posttest 1 (t1), and posttest 2 (t2)
- To measure changes in O2 Hb and HHb levels in the bilateral DLPFC at baseline, during, immediately after, and 20 minutes after 5 minutes each of S-C and S-T compared to 5 minutes of passive sitting
- To measure the cognitive test performance (n-back test and SCWT) and BP at baseline, immediately after, and 20 minutes after 5 minutes each of S-C and S-T compared to 5 minutes of passive sitting

The secondary objectives of the main study are as follows:

- To examine changes in cognitive performance (working memory and inhibitory control; n-back test and SCWT) from t0 to t1 and t2 under each intervention condition relative to its modality-matched control
- To examine changes in BP/heart rate (HR) from t0 to t1 and t2 relative to the modality-matched control
- To examine changes in self-reported daytime sleepiness and back pain from t0 to t2 under each intervention condition relative to its modality-matched control, as specified in the assessment schedule

#### Study Hypotheses

We hypothesize that compared with the modality-matched passive control, the 5-minute LIPA intervention will produce a greater t_0_-to-t_1_ change in DLPFC O_2_Hb during cognitive task performance (primary endpoint). We further hypothesize that O_2_Hb changes may persist at t_2_ and that cognitive performance (n-back test and SCWT accuracy/response time) may improve following the intervention (key secondary outcomes).

The hypotheses are as follows:

- Hypothesis (H)1 (hemodynamics): Compared with the modality-matched control (passive sitting), both S-C and S-T are associated with higher DLPFC O2 Hb (primary) and corresponding changes in HHb (supportive) during cognitive task performance at t1 and/or t2 relative to t0.
- H2 (cognition): Compared with the corresponding modality-matched control, both S-C and S-T will result in improved working memory and inhibitory control performance (eg, n-back test and SCWT outcomes) at t1 and/or t2 relative to t0.
- H3 (physiological and symptoms): Compared with the corresponding modality-matched control, both S-C and S-T will lead to favorable acute changes in BP/HR and reduced subjective daytime sleepiness and back pain at t2 relative to t0.

## Methods

### Ethical Considerations

The study was reviewed and approved by the Institutional Review Board (IRB) of Nanyang Technological University (approval number IRB-2023-939). All participants provided written informed consent prior to enrollment, and all procedures were conducted in accordance with the Declaration of Helsinki. Participation was voluntary, and participants could withdraw at any time without penalty. Data were collected and stored in a deidentified form on password-protected devices, with access restricted to the research team. Participants were compensated with cash payment (US $30 for full compliance) for their participation.

### Study Design

The trial was registered on November 21, 2024 (ClinicalTrials.gov identifier: NCT06703424), and participant recruitment commenced only after registration. This manuscript is presented as a protocol with trial status, describing the prespecified protocol together with the completed trial timeline/status.

Details of this protocol are reported in accordance with SPIRIT guidelines [34]. SPIRIT provides a comprehensive checklist to ensure clarity and completeness in trial protocol reporting, and the SPIRIT items addressed in this protocol are summarized in Table 1. Outcomes were assessed at baseline (t_0_), posttest 1 (t_1_), and posttest 2 (t_2_) to characterize both acute and short-term delayed effects of a brief LIPA bout. The immediate postintervention window was intended to capture rapid physiological and neurocognitive responses (eg, transient changes in prefrontal oxygenation and executive performance), whereas the 20-minute follow-up evaluated whether any effects persisted beyond the immediate period or attenuated toward baseline. The 20-minute interval was selected as a pragmatic postbreak window that fits within a typical study or class session and aligns with commonly used observation periods for short-lived responses after brief activity.

**Table 1:** Timeline of enrollment, interventions, and assessments in the main study.

SPIRIT^a^ items		Enrollment	Pre-intervention	During the intervention	Postintervention			
		–t_1_	t_0_^b^	t_during_^c^	t_1_^d^	t_2_^e^		
Enrollment								
	Eligibility screen	^✓^	—^f^	—	—	—		
	Informed consent	^✓^	—	—	—	—		
	Allocation (S-C^g^ or S-T^h^)	^✓^	—	—	—	—		
	Familiarization	—	^✓^	—	—	—		
Interventions								
	S-C or S-T	—	—	^✓^	—	—		
	Waitlist control	—	—	^✓^	—	—		
Assessments								
	fNIRS^i^	—	^✓^	^✓^	^✓^	^✓^		
	n-Back test	—	^✓^	—	^✓^	^✓^		
	SCWT^j^	—	^✓^	—	^✓^	^✓^		
	BP^k^	—	^✓^	—	✓^✓^	^✓^		
	Self-rating sleepiness	—	^✓^	—	—	^✓^		
	Self-rating back pain	—	^✓^	—	—	^✓^		

^a^SPIRIT: Standard Protocol Items: Recommendations for Interventional Trials.

^b^t_0_: baseline measurements (pretest).

^c^t_during_: time during the intervention (the intervention is 5 minutes long).

^d^t_1_: immediately after the exercise.

^e^t_2_: 20 minutes after t_1_.

^f^Not applicable.

^g^S-C: sit-cycling.

^h^S-T: stand-twisting.

^i^fNIRS: functional near-infrared spectroscopy.

^j^SCWT: Stroop Color and Word Test.

^k^BP: blood pressure.

### Study Setting and Participants

The trial was conducted at a university in Singapore. This manuscript is intended as a study protocol with trial status information, accompanied by supportive methodological evidence from a MET validation substudy. Prospective participants were students from the same university, aged between 21 and 30 years, who do not meet physical activity guidelines [36]. An a priori target sample size of 28 participants (n=14, 50%, per modality group) was estimated using G*Power 3.1 for a repeated-measures analysis with within-between factors (α=.05; 1 − β=0.80; Cohen f=0.25). Owing to recruitment constraints, 22 (78.6%) participants were ultimately enrolled (n=11, 50%, per modality group). This shortfall is acknowledged in the *Discussion* section as a limitation, with potential implications for statistical power, particularly for small-to-moderate effects. A suggested sample of 6 (27.3%) participants who met the selection criteria was recruited based on a G*power test (α=.05; power: S-C, 99%; S-T, 86%) for the substudy.

### Recruitment

For recruitment, study advertisements were displayed within the university campus and posted in student messaging groups on online messaging channels, such as WhatsApp and Telegram. Interested students contacted the research team, and a research team member explained the study’s purpose, the requirements for participation, and potential risks and benefits and addressed questions in person. Afterward, prospective participants were screened for eligibility. Those who met the inclusion criteria and were willing to participate signed an informed consent form that confirmed participation in the study.

The inclusion criteria were as follows:

- Age between 21 and 30 years
- Right-handed
- Physically inactive: less than 150 minutes of moderate-intensity physical activity (MPA) or less than 75 minutes of vigorous-intensity physical activity (VPA) per week in the past month [36]
- Cleared for LIPA based on the Get Active Questionnaire (GAQ) [37] (no positive responses on the GAQ indicating the need for medical clearance or medical clearance obtained, if required) and willing to undergo fNIRS
- Not taking any antihypertensive medication at present

The criterion of not meeting physical activity guidelines (<150 minutes of MPA or <75 minutes of VPA per week in the past month) was appraised by the researcher during recruitment based on each prospective participant’s response to the following questions [36]:

- How many days in a week do you engage in MPA or VPA?
- How many minutes on average per day do you engage in physical activity at this level?
- How many days per week do you engage in muscle-building or strength related exercise?

Examples and characteristics of MPA and VPA were shared in helping participants not familiar with MPA or VPA.

Reasons for exclusion included not meeting the physical inactivity criterion, not meeting safety criteria on the GAQ, or declining participation.

### Procedural Flow

**Figure 1:**
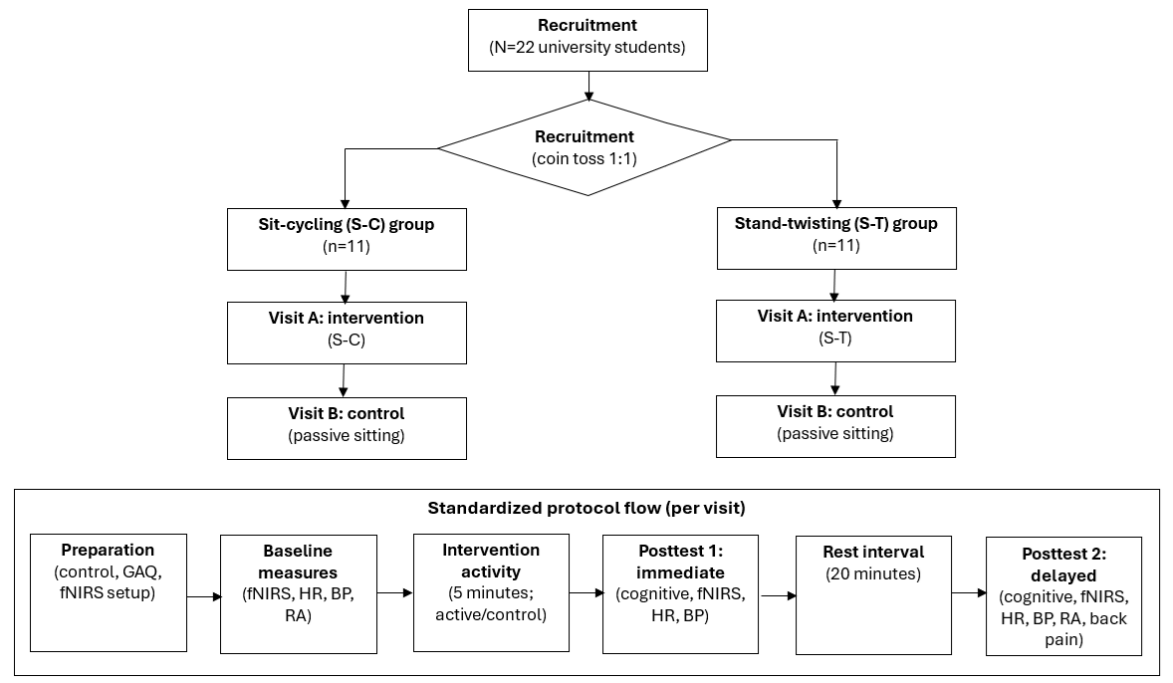
Revised study design and procedural flow. BP: blood pressure; fNIRS: functional near-infrared spectroscopy; GAQ: Get Active Questionnaire; HR: heart rate; RA: rating of alertness.

A screening log was maintained to document responses to advertisements, eligibility assessment outcomes, reasons for exclusion, and final enrollment. A total of 22 eligible participants provided written informed consent and were enrolled. Following allocation, each participant completed two laboratory visits within the assigned modality (an experimental LIPA visit and a modality-matched passive control visit), as shown in Figure 1. At each visit, outcomes were assessed at baseline (t_0_), during the 5-minute activity period (t_during_; intervention or control), posttest 1 (t_1_), and posttest 2 (t_2_). The number of participants completing each visit and each assessment (t_0_, t_during_, t_1_, and t_2_), as well as the number in final analytic sample, are presented in Figure 1.

### Data Collection Procedures: Assessment Time Points and Visit Structure

Within their assigned modality, each participant completed two laboratory visits (intervention and modality-matched passive control), forming a within-participant crossover for condition. As mentioned before, at each visit, outcomes were assessed at baseline (t_0_) after a 3-minute rest period, during the 5-minute intervention (t_during_), posttest 1 (t_1_), and posttest 2 (t_2_) 20 minutes after t_1_. Unless otherwise specified, all time points (t_0_, t_during_, t_1_, t_2_) refer to assessments during a visit. Subjective outcomes (sleepiness and back pain) were assessed only at t_0_ and t_2_ to minimize interruption during cognitive testing and to capture baseline and delayed symptom responses; they were not assessed at t_1_. Baseline measures (t_0_) included cognitive testing (n-back test and SCWT) and physiological measures (functional near-infrared spectroscopy [fNIRS], HR, BP, and rating of alertness [RA]). The intervention or control condition was delivered for 5 minutes, during which monitoring measures (eg, rating of perceived exertion [RPE] checks) were recorded (t_during_). The equipment included an S-C apparatus (AIBI EZ Tone Chair) and an S-T apparatus (AIBI Massage Body Twister) [38,39]. Immediately after the 5-minute bout, participants completed the immediate postintervention assessments (t_1_), including cognitive outcomes, fNIRS, HR, and BP measurements. This was followed by delayed assessments at t_2_ after a rest interval, which included cognitive outcomes, fNIRS, HR, BP, RA, and back pain. To minimize procedural variability, all sessions were conducted in the same laboratory setting using a standardized script and identical equipment setup, and outcome assessors followed a prespecified checklist for each time point.

### Protocol Design for the Substudy

**Figure 2:**
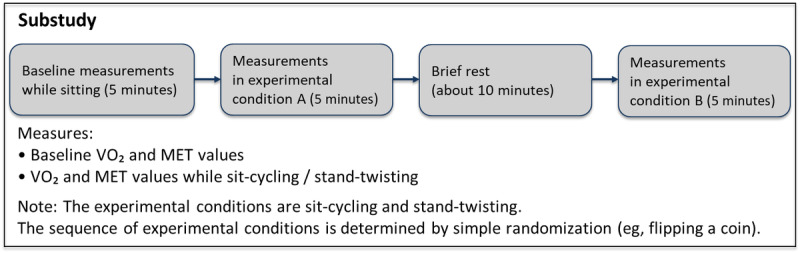
Schematic representation of the substudy. MET: metabolic equivalent; VO2: oxygen consumption.

A total of 6 (27.3%) participants for the substudy were drawn from the participant pool (N=22) for the main study. The 6 participants visited the exercise physiology laboratory on a designated day. Upon their arrival, a research team member explained the process to them. The participants were then allocated to either the S-C group or the S-T group, after which they spent approximately 5 minutes familiarizing themselves with either the S-C or the S-T equipment. During the indirect calorimetry measurement, a mouthpiece connected to a metabolic cart (Parvo Medics True One 2400) was attached to the participant’s head. The participant breathed through the mouthpiece while remaining seated for 5 minutes. The metabolic cart collected breath-by-breath expired air and recorded the rate of oxygen consumption (VO_2_) and METs. After baseline measurements were recorded, the participants rested for about 5 minutes. Subsequently, they underwent an indirect calorimetry session while doing S-C or S-T at a self-selected pace with minimal or negligible applied resistance for 5 minutes each. The total duration of the session averaged approximately 40 minutes. Figure 2 shows a schematic illustration of the substudy.

### Protocol Design for the Main Study

**Figure 3:**
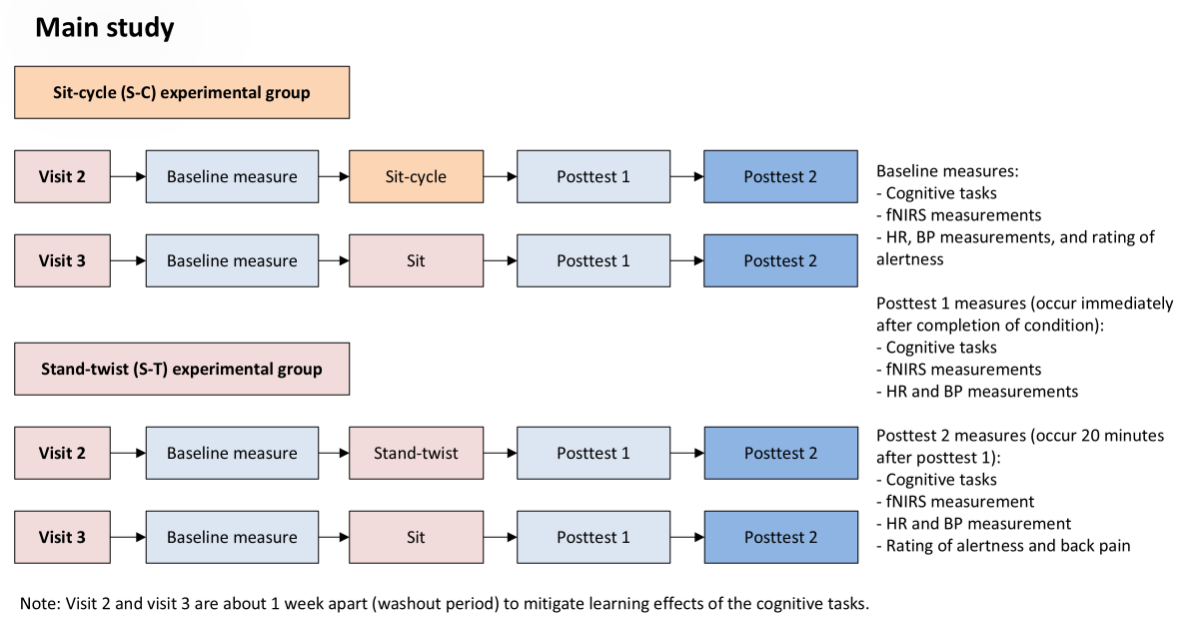
Schematic representation of the main study. BP: blood pressure; fNIRS: functional near-infrared spectroscopy; HR: heart rate.

Participants were allocated by the researcher to either the S-C or the S-T group in a 1:1 ratio using a coin toss for the first assignment (random start), followed by a prespecified alternating allocation sequence based on enrollment order. Within each assigned modality, outcomes were assessed repeatedly across prespecified time points (t_0_, t_1_, t_2_) and across two occasions (the experimental LIPA condition and the corresponding modality-matched passive control condition). As allocation concealment was not implemented and subsequent assignments were predictable, this approach is described as sequential (quasi-random) allocation rather than full randomization. Figure 3 shows the timeline of enrollment, intervention, and assessment phases of the main study.

### Intervention

#### S-C Experimental Condition

Participants assigned to the S-C intervention group cycled on the EZ Tone Chair at a self-selected cadence against a minimal applied resistance for 5 minutes. The chair measures 57 × 39 × 76 cm^3^ in dimension and is equipped with toe cap foot pedals. Previous research in the same institution used it as an exercise tool for reducing sedentary behavior in desk-bound participants [33]. In addition, participants were asked to rate their exertion levels on the 1-10 Borg RPE scale at every 1-minute interval.

#### S-T Experimental Condition

Participants assigned to the S-T intervention group stood on the AIBI Massage Body Twister and twisted their torso along the transverse plane in an alternating clockwise and counterclockwise direction with even arm swings for balance and at a comfortable cadence (about 2 seconds for a full left-right trunk twist) for 5 minutes. The twister is a compact piece of equipment with a diameter of 35 cm and is portable, making it convenient for use in any setting. It has been used as an intervention method for reducing sitting time and investigating the weight loss effects in participants with obesity [40]. In addition, participants were asked to rate their exertion levels on the 1-10 Borg RPE scale at every 1-minute interval.

### Intervention Delivery and Fidelity

Interventions were delivered under direct supervision of a trained research team member to ensure standardization and participant safety. Both S-C and S-T groups received the same brief standardized instructions regarding posture, cadence, and safety before they started the 5-minute bout. Perceived exertion was recorded at 1-minute intervals using the Borg RPE scale to monitor intensity and support fidelity to LIPA.

Participants in the control groups spent a modality-matched passive control period with no structured physical activity (passive sitting on a chair or standing still) for the same 5-minute duration. Outcome assessments were conducted at identical time points as in the interventions. The modality-matched passive control conditions were selected to match the session duration and posture as closely as possible; however, they were not designed as sham interventions and therefore may not have adequately controlled for posture-related arousal, expectancy effects, or other context-related cues (eg, awareness of movement and perceived exertion).

Any protocol deviations and adverse events were documented.

### Key Outcome Measures

#### Primary and Secondary Outcomes

The primary endpoint of the main trial is the within-person intervention-control difference in the change in O_2_Hb in the bilateral DLPFC during cognitive task performance from baseline (t_0_) to posttest 1 (t_1_). The DLPFC O_2_Hb metric is defined as the bilateral DLPFC region-of-interest average, calculated as the baseline-corrected mean O_2_Hb during cognitive task performance at each time point.

Key secondary outcomes include (1) persistence of the O_2_Hb response posttest 2 (t_2_), (2) cognitive performance on the n-back test and the SCWT (accuracy and response time, as applicable), (3) HHb in the same DLPFC region (supportive physiological indicator), (4) BP and HR, and (5) symptom-based outcomes (eg, subjective daytime sleepiness and back pain measures).

#### Functional Near-Infrared Spectroscopy

**Figure 4:**
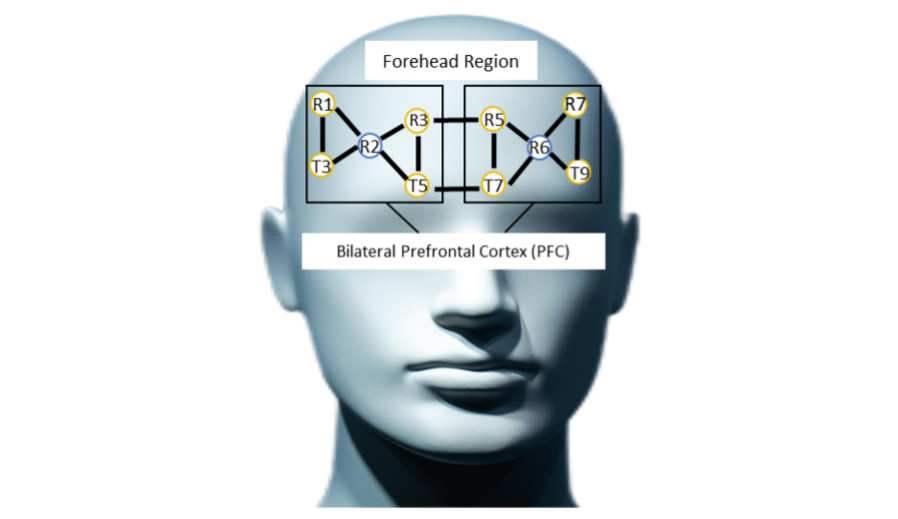
Illustration of the optode montage used indicating brain measurements using fNIRS (blue color: transmitters; yellow color: receivers). fNIRS: functional near-infrared spectroscopy.

fNIRS is based on the principle that most biological tissues are transparent to near-infrared light (NIR), while O_2_Hb and HHb better absorb NIR light in the 700-900 nm spectrum. fNIRS uses the modified Beer-Lambert law to measure the absorption rate of these hemoglobin species at two different NIR wavelengths between transmitter and receiver pairs placed on the scalp. In this study, an eight-channel portable continuous-wave NIRS system (Octamon, Artinis Medical Systems) was used to measure fNIRS signals during the n-back test and the SCWT. Hemodynamic responses were recorded from the bilateral DLPFC using eight transmitters and two receivers (one receiver receiving from four transmitters on each hemisphere; see Figure 4) over the forehead region, with each receiver measuring time-multiplexed NIR light from four surrounding transmitters that were 3 cm apart, resulting in eight data channels. All signals were sampled at 10 Hz. A MATLAB-based toolbox was used to accurately estimate the spatial brain mapping of the electroencephalography (EEG) 10-20 system onto associated Brodmann areas, identifying the left and the right DLPFC as the regions of interest. Following data collection, the fNIRS data will be processed using the HOMER3 MATLAB toolbox with the following pipeline: (1) conversion to optical density changes, (2) removal of low signal-to-noise channels, (3) motion artifact detection and correction, (3) bandpass filtering, and (4) conversion to concentration changes using the modified Beer-Lambert law, with block averaging to obtain the average change across three trials for each cognitive task condition.

#### n-Back Test

The n-back test is a cognitive assessment tool used to evaluate working memory capacity and cognitive inhibition [41]. It has also been widely used as a training tool for enhancing working memory [42,43]. During the n-back test, participants were shown a sequence of visual stimuli in the form of digits from 0 to 9 and were asked to indicate whether the current stimulus shown matched the one presented n characters earlier by pressing the designated letters on the keyboard (eg, letter “F” to indicate a match and “J” to indicate a mismatch). For this study, the 1-back and 2-back tests were adopted. In the 1-back test, participants were required to recall and indicate whether the current digit shown matched the previous digit, whereas in the 2-back test, participants indicated whether the current digit shown matched the second-last digit presented earlier. The n-back test consists of three blocks of 1- and 2-back tasks. A practice block was added to the baseline pretest, where participants were given 20 stimuli (letters) to familiarize themselves with the process before moving on to the actual test. The actual test consisted of three blocks with 30 stimuli (letters). The test began with a centered fixation cross on-screen for 500 ms, followed by the stimulus in that location for 1000 ms, followed by a 500 ms interstimulus interval. The *accuracy* (number of correct recalls) and *response time* (speed of recall) were assessed at the end of each block. The study design entailed participants to undergo the n-back test thrice (baseline, posttest 1, and posttest 2) on two occasions (experimental condition and waitlist control condition). Changes in the n-back test score from baseline to posttests 1 and test 2 were measured.

#### Stroop Color and Word Test

The SCWT is a cognitive test widely used to assess the ability of an individual to inhibit cognitive interference that occurs when the processing of a specific stimulus feature is impeded by the simultaneous processing of another attribute of the same stimulus [44]. In this study, the test consisted of a series of color words (eg, “red,” “blue,” “green,” and “yellow”) printed in colored ink, with the font color sometimes matching the word and sometimes not. We adopted the incongruent condition, where participants were shown a sequence of stimuli in which the word text matched or did not match the color of the ink (eg, the word “red” could be printed in blue ink). Participants were required to indicate whether the word text matched the color of the ink by pressing the designated letter on the keyboard (eg, letter “F” to indicate a match and “J” to indicated a mismatch). The color of the words was displayed on the screen one at a time for 2000 ms. Participants were presented with six blocks of words, alternating between congruent and incongruent words. The test typically consists of multiple blocks (three or four) and records the *accuracy* of each response (number of correct texts and colors) and the *reaction time* (time taken to input a response). A practice block was added to the baseline pretest for familiarization like in the n-back task. Participants underwent the test thrice at different time points (ie, baseline, posttest 1, and posttest 2) on two occasions (experimental condition and waitlist control condition), and changes in the SCWT scores from baseline to posttests 1 and test 2 were determined.

#### BP and HR Measurement

Participants’ BP and HR were measured using the Vivo Watch BP HC-A04 (ASUSTek Computer Inc) worn on the wrist. With a pair of electrocardiography (ECG) and photoplethysmography sensors embedded in the watch, the Vivo Watch BP HC-A04 provides systolic and diastolic BP and HR readings. Previous studies have evaluated the validity of smartwatch-based BP-monitoring features, and validation evidence has also been reported for an ASUS VivoWatch series device against reference sphygmomanometer measurements [45]. Accordingly, BP readings in this study will be interpreted primarily for research monitoring (ie, within-participant comparisons across time points) rather than for clinical diagnosis. Measurements of BP and HR were taken at baseline, posttest 1, and posttest 2, and changes in the BP and HR readings from baseline to posttests 1 and 2 were determined.

#### Stanford Sleepiness Scale

The Stanford Sleepiness Scale (SSS) is a self-rating tool designed to evaluate changes in sleepiness on a 7-point scale, applicable at any time of day [44,46]. In this study, participants were asked to rate their level of sleepiness upon visiting the test site at baseline and posttest 2 by choosing a number and description that best described their state. The scale ranges from “feeling active, vital, alert, or wide awake” to “no longer fighting sleep, sleep onset soon, or having dream-like thoughts.” The SSS correlates well with task performance related to alertness [47], and it has been previously used to gather subjective sleepiness data among workers [48].

#### Oswestry Low Back Disability Questionnaire

The Oswestry Low Back Disability Questionnaire (OLBDQ) is a widely used tool for assessing functional abilities relating to low back pain. The self-reported questionnaire has been validated and its reliability tested, and it is used in different populations [49]. In this study, participants completing the OLBDQ were asked about how low back pain affects their ability to manage their everyday life. The different contexts in everyday life included in the questionnaire are (1) pain intensity, (2) personal care (washing, dressing), (3) lifting objects, (4) walking, (5) sitting, (6) standing, (7) sleeping, (8) social life, and (9) traveling. For example, regarding pain intensity, participants were asked to select the statement that best described their problem: “I have no pain at the moment,” “The pain is very mild at the moment,” “The pain is moderate at the moment,” The pain is fairly severe at the moment,” “The pain is very severe at the moment,” or “The pain is the worst imaginable at the moment.” For each section, the total possible score is 5, and each statement is marked with a score; for example, the first statement is marked with a section score of 0, the second statement is marked with a section score of 1, and so on until the sixth statement is marked with a section score of 5. There are nine application sections, yielding a total possible score of 45. A higher score is interpreted as more severe low back functional disability. In this study, the OLBDQ was administered at t_0_ and t_2_ during each visit to capture baseline and delayed responses; it was not administered at t_1_.

### Blinding

Owing to the behavioral nature of the intervention conditions, blinding of participants and intervention personnel was not feasible. To minimize performance and detection bias, all sessions follow a standardized protocol with scripted instructions, and objective outcomes were obtained using instrument-based or computer-administered procedures (eg, fNIRS-derived hemodynamic responses and computerized cognitive tasks). Data processing and analyses will follow prespecified procedures.

### Statistical Analysis

Descriptive statistics will be reported as the mean (SD) for approximately normally distributed continuous variables and as the median (IQR) for skewed variables, while categorical variables will be summarized as counts and percentages. The primary endpoint is the within-person intervention-control difference in the change in bilateral DLPFC O_2_Hb levels during cognitive task performance from baseline (t_0_) to posttest 1 (t_1_), while HHb will be analyzed as a supportive indicator. The DLPFC O_2_Hb metric will be the bilateral DLPFC region-of-interest average (baseline-corrected mean O_2_Hb during the cognitive task at each time point). The primary analysis will test the within-person intervention-control difference in the change in DLPFC O_2_Hb from t_0_ to t_1_ (primary endpoint) using a prespecified linear mixed-effects model, with participant as a random effect and condition (intervention vs control), time, and assigned modality (S-C vs S-T) as fixed effects, including condition × time (and, where relevant, condition × time × modality) interaction terms. The primary endpoint will be evaluated at a two-sided α=.05. For key secondary outcomes (O_2_Hb persistence at t_2_ and cognitive performance measures), we will apply Holm-Bonferroni adjustment across the prespecified key secondary tests (O_2_Hb persistence at t_2_ and the prespecified cognitive performance endpoints [accuracy and response time, as applicable]); all other outcomes will be interpreted as secondary/exploratory. The visit order/session will be included as a covariate to address potential practice or order effects. The primary contrast of interest will test the condition difference in the change from t_0_ to t_1_ (ie, condition × time contrast corresponding to t_0_→t_1_). Prespecified secondary contrasts will examine persistence at t_2_ (eg, t_0_→t_2_). Model assumptions will be assessed using standard diagnostics (eg, residual distributions and influence). Because the large multimodal model (LMM) is used for primary analyses, no sphericity assumption is required. Missing data will be handled using maximum likelihood estimation in the LMM under a missing-at-random assumption; the extent and patterns of missingness will be described. For secondary outcomes (cognitive test performance, BP, subjective sleepiness, and back pain), analogous LMMs will be fitted using the same modeling framework (participant as a random effect; condition, time, and modality as fixed effects, as appropriate). To address multiplicity across key secondary outcomes, we will control the family-wise error rate using a Holm-Bonferroni procedure, while maintaining a two-sided α of .05 for the primary endpoint. Where distributional concerns remain, nonparametric analyses (eg, Friedman test, Wilcoxon signed-rank test, Mann-Whitney U test, as appropriate) will be used as sensitivity analyses to assess robustness. Correlation analyses will use Pearson’s r for approximately normally distributed variables and Spearman’s ρ otherwise. Comparisons of outcomes by sex will be treated as exploratory and not powered, given the sample size (N=22), and will be reported with effect sizes and 95% Cis, where applicable. All tests will be two-sided. The primary endpoint will be evaluated at α=.05; key secondary outcomes will be interpreted using Holm-Bonferroni–adjusted inference, and all other analyses will be considered exploratory.

## Results

### Trial Status

The study protocol was approved by the IRB of Nanyang Technological University (approval number: IRB-2023-939) and was funded by the Sendai University International Collaborative Research Grant (grant number: CER-2023) in April 2023. Recruitment and data collection were completed in December 2024. A total of 22 physically inactive university students were enrolled and allocated to either the S-C or the S-T arm, with outcomes compared against the corresponding modality-matched passive control condition (passive sitting for S-C and S-T). Data cleaning and preliminary analyses are ongoing, and the complete outcomes will be reported in a subsequent results paper (anticipated in late 2026).

### Methodological Substudy Results: MET Validation for Intervention Intensity

A methodological substudy was conducted to confirm that the two 5-minute activity modalities (S-C and S-T) meet the operational definition of LIPA (<3 METs), thereby justifying their classification for the main trial. The mean MET value was 0.98 during 5 minutes of quiet seated rest during the modality-matched passive control stage and 2.71 during S-C (paired *t* test, *P*<.05), indicating higher energy expenditure during S-C than during seated rest. Similarly, the mean MET value was 1.19 during 5 minutes of quiet standing and 2.26 during S-T (paired *t* test, *P*<.05), indicating higher energy expenditure during S-T than during quiet standing. As a safety check, paired-sample *t* tests showed no significant pre-post differences in BP or HR following either 5-minute bout. Collectively, these findings indicate that both 5-minute S-C and S-T bouts fell within the LIPA range (<3 METs) and could be implemented as brief, standardized LIPA breaks in the main trial. No analyses of the main trial outcomes (fNIRS-derived prefrontal cortex hemodynamics, cognitive performance, BP, subjective sleepiness, and back pain) are reported in this protocol paper; these results will be presented in a separate outcomes manuscript once analyses are finalized.

## Discussion

### Summary

This manuscript primarily serves as a study protocol with trial status information, detailing the rationale, design, procedures, and planned analyses for examining the acute effects of two 5-minute LIPA breaks (S-C and S-T) on neurocognitive and somatic outcomes among physically inactive university students. In addition, we reported findings from a methodological substudy undertaken to support the main trial by confirming that both S-C and S-T can be operationalized as LIPA (<3 METs). Taken together, this protocol provides a transparent framework for replication and for interpreting subsequent outcomes reporting from the main trial.

The primary aim of this protocol was to establish that a 5-minute bout of S-C or S-T constitutes LIPA. Consistent with our hypothesis, 5 minutes of both S-C and S-T were deemed as LIPA. These findings provide methodological support for the main study, which is designed to investigate the acute effects of LIPA on cognitive function and brain activity. Establishing the intensity of these modalities is an essential methodological step, as the primary trial aims to evaluate whether a brief LIPA bout can elicit measurable changes in prefrontal cortex hemodynamics and cognitive performance, alongside changes in subjective sleepiness and back pain. If effective, such short activity breaks may represent a pragmatic strategy to interrupt prolonged sitting in student populations who are at elevated risk of sustained sedentary exposure.

From a broader perspective, the importance of interrupting prolonged sitting, even among physically active individuals, has been emphasized [50]. In parallel, interventions using digital health technologies to reduce sedentary behavior in workplaces and to promote behavior change are gaining popularity [51]. Although much of the existing evidence on these digital interventions focuses on general health outcomes, evidence relating to brain activity and cognitive function remains limited. Research investigating how short physical activity breaks influence cerebral blood oxygenation and cognitive performance in adults is still in its infancy, and key parameters, such as the optimal exercise modality, bout duration, and interruption frequency, that are feasible in real-world settings are yet to be established. In this context, by integrating objective indices (eg, prefrontal cortex oxygenation) with behavioral and symptom-based outcomes (eg, working memory, inhibitory control, sleepiness, and back pain), this trial is expected to contribute to a more integrative understanding of how short, low-intensity movement may influence both neurocognitive and musculoskeletal-related indicators in young adults. Moreover, this protocol provides a practical framework to inform future studies seeking to elucidate whether brief S-C and S-T breaks, compared with prolonged sitting, can meaningfully reduce sedentary exposure and support cognitive functioning and overall well-being.

This protocol is underpinned by the MEF, which provides a robust foundation for understanding the multifaceted role of movement in health promotion. By valuing all movement behaviors, including LIPA, the MEF encourages a holistic approach to health that may be particularly relevant for university students, who are vulnerable to sedentary lifestyles [6,52].

### Strengths and Limitations

The strengths of this protocol include the pragmatic nature of the intervention (brief, low-cost, and implementable in common study settings), clearly specified eligibility criteria, standardized delivery of activity bouts, and prespecified outcome assessment time points. These features are intended to enhance transparency and reproducibility and to facilitate translation into real-world university contexts, such as classrooms, libraries, and other desk-bound study spaces.

A further strength of this protocol is the assessment schedule incorporating immediate and short-term follow-up. By evaluating outcomes at t_0_, t_1_, and t_2_, the study can differentiate transient responses from effects that persist for a meaningful period within a typical academic session, thereby improving interpretability and translational relevance.

Several limitations should be acknowledged when interpreting this protocol and the subsequent outcomes reporting. First, the achieved sample size (N=22) was smaller than the a priori target (N=28), which may reduce statistical power, particularly for detecting small-to-moderate effects. Accordingly, findings should be interpreted cautiously, with emphasis on effect sizes and Cis, in addition to statistical significance. Second, this is a nonrandomized study in which modality assignment followed a sequential (quasi-random) alternating schedule and allocation concealment was not implemented; therefore, a risk of selection bias cannot be ruled out despite consecutive enrollment and the use of a prespecified allocation schedule. In addition, because the protocol involves brief behavioral tasks assessed repeatedly over time, performance-related influences (eg, differential engagement, learning, or fatigue across repeated assessments) may affect outcomes, particularly for subjective measures, such as sleepiness and back pain. Third, blinding was not feasible for participants or intervention personnel due to the nature of the activity conditions, which may increase susceptibility to expectancy and performance effects, particularly for subjective outcomes, such as sleepiness and back pain. Although objective outcomes (eg, prefrontal cortex hemodynamics and computerized cognitive task performance) are relatively less susceptible to reporting bias, nonblinding may still influence participant engagement and effort during assessments. Fourth, although the analytical plan specifies a primary endpoint and prespecified multiplicity control for secondary outcomes, the assessment of multiple outcomes still warrants cautious interpretation to mitigate the risk of inflated type I errors. Finally, as the study was conducted within a single university context, generalizability may be limited.

### Future Directions

Future research should build on this protocol by implementing a fully randomized allocation procedure with allocation concealment; incorporating a sham or active control condition that better matches posture and expectancy; and recruiting larger samples, preferably across multiple sites, to improve precision and generalizability. In addition, preregistering a single primary endpoint (or a clearly ranked hierarchy of outcomes), applying a priori multiplicity control strategies, and considering linear mixed modeling approaches for repeated measures would further strengthen inferential robustness. Longer-term and ecologically embedded evaluations (eg, repeated breaks across a study day or semester) may also clarify whether acute responses translate into sustained benefits in cognitive performance and musculoskeletal symptoms.

## Abbreviations

BP: blood pressure
DLPFC: dorsolateral prefrontal cortex
fNIRS: functional near-infrared spectroscopy
GAQ: Get Active Questionnaire
Hhb: deoxygenated hemoglobin
HR: heart rate
IRB: Institutional Review Board
LIPA: light-intensity physical activity
LMM: large multimodal model
MEF: Movement Ecology Framework
MET: metabolic equivalent
MPA: moderate-intensity physical activity
NIR: near-infrared light
O₂Hb: oxygenated hemoglobin
OLBDQ: Oswestry Low Back Disability Questionnaire
RA: rating of alertness
RPE: rating of perceived exertion
S-C: sit-cycling
SCWT: Stroop Color and Word Test
SPIRIT: Standard Protocol Items: Recommendations for Interventional Trials
SSS: Stanford Sleepiness Scale
S-T: stand-twisting
VO2: oxygen consumption
VPA: vigorous-intensity physical activity

## Data Availability

Research data collected in this study will be deposited to the data repository of the authors’ university, as required by the university.
